# Evaluation of Wellby, a Cocreated Mobile App and Wearable to Support Stress Management and Overall Well-Being: Mixed Methods Acceptability and Usability Study

**DOI:** 10.2196/79381

**Published:** 2026-04-09

**Authors:** Justin Laiti, Sina Javadpour, Jenna Mullen, Claire Conway, Elaine Byrne, Pádraic Dunne

**Affiliations:** 1 Centre for Positive Health Sciences, Royal College of Surgeons in Ireland, 123 St Stephen's Green, Dublin, D02 YN77, Ireland, 353 1 402 2100; 2 Tissue Engineering Research Group, Royal College of Surgeons in Ireland, Dublin, Ireland; 3 School of Medicine, Royal College of Surgeons in Ireland, Dublin, Ireland

**Keywords:** adolescents, stress, sleep, wearable electronic devices, mobile apps, cocreation, acceptability, technology acceptance model, TAM

## Abstract

**Background:**

Digital well-being support tools can offer adolescents tailored interventions embedded in their digital environments. However, there is a lack of high-quality, evidence-based digital interventions specifically designed for young people’s well-being needs. Wellby is a mobile app and wearable device cocreated with Irish secondary school students to support stress management and overall well-being.

**Objective:**

This study aimed to evaluate the acceptability and usability of the Wellby system using the technology acceptance model, focusing on ease of use, perceived usefulness, and behavioral intention among Irish secondary school students.

**Methods:**

A mixed methods acceptability and usability study was conducted with Irish secondary school students (n=43) across 3 schools: 2 mainstream secondary schools and 1 Youthreach center for early school leavers. Students accessed the Wellby mobile app for 8 weeks and received the custom wearable device for the final 4 weeks (April-May 2024). Wellby included mood tracking, to-do lists, educational resources, text-based health coaching, and heart rate variability biofeedback with guided breathing exercises. Acceptability and usability were evaluated using the user version of the Mobile App Rating Scale (uMARS), structured focus groups, and app engagement analytics. Secondary measures included baseline well-being questionnaires (Perceived Stress Scale, Pittsburgh Sleep Quality Index, and EPOCH Measure of Adolescent Well-Being).

**Results:**

Among survey respondents (n=29), uMARS subscale scores were high: information quality (mean 4.2/5.0), functionality (4.1/5.0), and aesthetics (4.1/5.0). The perceived impact subscale showed the highest scores for well-being awareness (3.8/5.0) and help-seeking intention (3.9/5.0). Of survey respondents who participated in prior co-design sessions, 88% (21/24) and 83% (20/24) felt the wearable and app, respectively, aligned with their co-design feedback. App engagement more than doubled following the introduction of the wearable and revealed that the wearable tab was most accessed (643/1688, 38% of interactions), followed by the home (430/1688, 25%), resources (338/1688, 20%), and coaching (277/1688, 16%) tabs. Focus group feedback highlighted the mood tracker as the most valued feature and identified technical improvements, including better battery life, enhanced Bluetooth connectivity, and more personalization options.

**Conclusions:**

This study demonstrates that cocreated digital well-being tools can achieve high acceptability and usability with Irish secondary school students. Applying the technology acceptance model effectively captured student experience through mixed methods feedback. Students particularly valued self-tracking and personalization features over coaching or educational features. These findings highlight the importance of aligning digital technologies with adolescents’ needs and preferences, such as features that encourage increased autonomy and identity formation. Future iterations of Wellby should address the technical limitations while continuing the involvement of students to adapt to their developmental needs and maintain high acceptability.

## Introduction

### Background

Digital tools present novel pathways to enhance the health and well-being of young people, particularly given the ubiquity of digital technology in many adolescents’ lives [1]. Mobile apps offer scalable and accessible routes for adolescents to avail themselves of educational tools and interventions that address their well-being needs [2]. These can facilitate healthy lifestyle habits during the formative period of adolescence, which can encourage positive health later in life and reduce the burden of preventable noncommunicable diseases [3,4]. Additionally, connected technologies can augment the well-being support capabilities of mobile apps, such as wearable devices, which are increasingly used for tracking activities, health behaviors, and continuous physiological data [5]. While mobile apps include affordances that show promise in supporting adolescent well-being, there is limited research into adolescent-specific digital tools [6,7]. Given the potential for these tools to provide crucial support for young people, there is a need to further investigate and develop digital tools that are specifically designed to consider the health and well-being needs of this age group [2,8]. Because social, environmental, and demographic factors significantly impact adolescent health outcomes [9,10], understanding local contexts and cultures is critical for designing tailored interventions for specific cohorts of adolescents [7,11]. Several studies have demonstrated the importance of adopting adolescent-centered designs to promote intervention acceptability, usability, and effectiveness [12-15].

### Co-Design and Participatory Approaches

Participatory design practices are well documented throughout history [16], and there are several modern frameworks for engaging participants in the cocreation of new interventions with young people [17,18]. Radical participatory design is one framework for any population that stresses the need to shift power from researchers or designers to the participants throughout each phase of the intervention creation process [16]. While many design processes involve users only after a prototype has been developed, co-design and cocreation offer a more inclusive approach by positioning participants as equal partners throughout the entire process [19]. The term co-design tends to focus solely on the technology development process, while cocreation includes broader involvement of participants such as during planning and implementation phases [19]. This collaborative approach is particularly important when designing for adolescents, as their unique contexts and preferences require specialized consideration.

When developing digital tools for this age group, it is also essential to acknowledge the risks of these technologies so that potential privacy and safety concerns are considered in the design. Digital tools present several potential risks, including concerns about data privacy, inappropriate content exposure, overuse or dependence, algorithmic bias, and insufficient safeguarding mechanisms [20,21]. Specifically, an increased reliance on mobile apps, particularly social media, has been associated with increased anxiety and depression among young people [22,23]. Often, these apps implement addictive features, aiming to maximize people’s time on the platform, which can be particularly harmful for adolescents [24]. Participatory engagement and acceptability testing can help to mitigate these risks by centering the experiences, input, and impact on the people who will use these technologies.

### Current Gaps in Adolescent Digital Health

Despite the potential of digital tools to improve adolescent health [1,6,7], there remains a gap in evidence-based digital interventions focused on supporting adolescent well-being. A review of over 1000 mobile apps available for wellness and stress management found that only 2% were supported by scientific research papers, while 4% of the apps focused on either young adults or youth [25]. Similarly, reviews of mobile apps targeting adolescents have found a need for more rigorous evaluation and the incorporation of co-design processes to promote relevant and engaging interventions for young people [6,26]. Among platforms co-designed with young people, many focus on chronic disease management or clinical mental health conditions rather than stress or overall well-being [17].

In response to the lack of adolescent-specific and evidence-based interventions, researchers have increasingly called for more rigorous evaluation of these tools to better understand their effectiveness, safety, and long-term impact on adolescent well-being [7,27,28]. Fields such as positive technology have emerged to focus on the minimization of risk while maximizing features that promote healthy and meaningful engagement with technology [29]. Additionally, participatory design processes can help to mitigate risks by fostering trust between adolescents, caregivers, and researchers or developers, while also ensuring alignment with the adolescents’ goals and preferences [30].

### Related Acceptability and Usability Studies

The assessment of acceptability and usability can help inform the iterative development of technology [31] and enhance the impact on participants’ well-being [32]. This can be measured in several ways, including quantitative assessments using validated instruments, engagement patterns captured through analytics, and qualitative feedback on people’s experience with technology. Inal et al [31] conducted a systematic review of usability evaluations for mobile mental health technologies, analyzing 42 studies across various populations. In this review, they found that data were most often collected through questionnaires, field studies, and interviews, with user reception and people’s perception of the intervention being the main outcomes of focus. Notably, only 3 of the 42 included studies incorporated participatory design in the development of the technologies that were tested. Another systematic review by Creaser et al [33] synthesized results from 33 studies on the acceptability of wearable activity trackers for promoting physical activity in youth. This found that wearables increased motivation in young people to be more physically active, despite technical difficulties and novelty effects of wearables use.

A similar study investigated the app engagement, usability, and satisfaction of adolescents, aged 13‐20 years, interacting with the BeMe app, focused on supporting adolescent mental health [34]. The BeMe app included content based on cognitive behavioral therapy, interactive activities, text-based coaching, and links to clinical services. The majority of participants in this study engaged with the content, mood ratings, and skill training activities on the app, and greater participant engagement was associated with increased app satisfaction. Another study assessed the usability and acceptability of Fitbits for Australian adolescents aged 13‐14 years during a 6-week period [35]. The adolescents in this study found the Fitbit easy to use and useful for monitoring physical activity, with barriers including comfort, design issues, and lack of activity-specific feedback. Additionally, Martin et al [36] outlined the co-design of a mobile app to promote a healthy lifestyle in European adolescents aged 13‐16 years. Based on the co-design process, a series of recommendations were established for the mobile app including personalization features, accessible language and design, and comprehensive app tutorials. Finally, Swahn et al [37] summarized feedback from female adolescent participants using a wearable in an urban setting in Uganda. Despite general positive feedback from participants, they also shared concerns about comfort, surveillance, and data privacy, and strong reactions from community members. Although these studies focus on adolescent feedback on digital well-being tools, most evaluate either mobile apps or wearable devices in isolation.

Furthermore, few studies employ theoretical frameworks to systematically assess the acceptability and usability of these tools. The technology acceptance model (TAM) was chosen for this study as it offers a simple yet effective and adaptable framework for acceptability that has been applied in diverse contexts, such as education [38]. Other common frameworks include the unified theory of acceptance and use of technology (UTAUT) [39] and the theoretical framework of acceptability (TFA) [40]. The UTAUT is based on several frameworks, including the TAM, and outlines 4 key factors and modifiers that influence intention and usage, while the TFA was established for health-related interventions and includes prospective, concurrent, and retrospective components of acceptability. The TAM offers several advantages for this study. It presents a robust measure with previous application in similar adolescent and educational contexts [38,41], enabling comparison to related work and promoting reproducibility and adaptability of this method for future studies.

### Aim and Hypothesis

This paper outlines student evaluations of the acceptability and usability of Wellby, a cocreated mobile app and wearable, to help to iteratively develop this intervention for student well-being. The acceptability and usability testing detailed in this paper helps to maintain alignment with students’ needs and promote beneficial technology usage. The mixed methods approach of this study is transferable to other cocreated interventions, and students offer feedback that can be used to enhance digital tools more generally to support adolescent-specific well-being needs.

The aim of this study is to assess the acceptability and usability of the Wellby mobile app and wearable as part of a cocreation process with Irish secondary school students. The primary outcomes are ease of use, perceived usefulness, and behavioral intention of Wellby, which are measured using the TAM framework both quantitatively and qualitatively [42]. Secondary outcomes are students’ stress, sleep, and overall well-being, measured by baseline questionnaires and demographic information to further contextualize the results. Wellby has been created through an iterative cocreation process with students (aged 15‐19 years) at 4 Irish secondary schools to address students’ well-being needs through a mobile app and wearable that offer educational resources, heart rate variability (HRV) tracking, and text-based coaching. Based on participatory design principles, we hypothesize that the cocreation process will lead to a system that aligns with students’ well-being goals and demonstrates high acceptability and usability among students. This study was conducted across 3 of the 4 Irish secondary schools that were involved in the co-design process. Acceptability and usability feedback was gathered to adapt the next version of Wellby to provide relevant stress and well-being management tools for students.

## Methods

### Overview

This mixed methods acceptability and usability study evaluated Wellby over 8 weeks with Irish secondary school students (n=43) based on the TAM [42].

### Ethical Considerations

Ethical approval for this study was obtained through the Research Ethics Committee at the Royal College of Surgeons in Ireland (ID 202403004). Informed consent was obtained from students’ parents and/or legal guardians, with written assent provided by adolescents. This study was registered with ClinicalTrials.gov (NCT06294210). Participant data were pseudonymized, and qualitative feedback was assessed for identifiable information to preserve participant privacy. No compensations or incentives were provided to participants besides the potential benefits of the intervention itself.

### Participants

Students from 3 schools participated in this study. Two of the schools were mainstream schools, while one school was a Youthreach center. Youthreach is an alternative coeducational program with locations around Ireland, providing specialized education for early mainstream school leavers [43]. The participants at the mainstream schools (schools A and B) were in their 5th year, aged 16‐17 years. School A is a coeducational school in County Wexford, and school B is an all-female school in County Kildare. The survey participants at Youthreach (school C), in County Dublin, were in their first or second year of the program, aged 15‐19 years. Eighty-eight percent of student participants were also involved in previous co-design sessions (Table 1). In usability studies, a baseline of 5‐10 participants is recommended by Macefield [44]. This target number was reached for each school in this study to ensure representative feedback since these schools previously showed slight differences in well-being needs [45].

**Table 1.** Student participant demographics by school.

Characteristics	School A (n=16), n	School B (n=11), n	School C (n=16), n	Total (n=43), n
Age (years)				
16	5	2	5	12
17	11	7	7	25
18	0	2	1	3
19	0	0	3	3
Race and ethnicity				
White Irish	12	5	13	30
White/other European	4	2	1	7
Asian	0	2	1	3
Black or Black Irish	0	1	1	2
White African	0	1	0	1
Gender				
Female	8	10	9	27
Male	8	0	5	13
Nonbinary	0	0	1	1
Prefer not to say	0	1	1	2
Previous research engagement				
Prior co-design participants	13	10	15	38
New participants	3	1	1	5
Wearable user				
Yes	3	1	2	6
No	13	10	14	37

### Wellby Mobile App and Wearable Description

**Figure 1.**
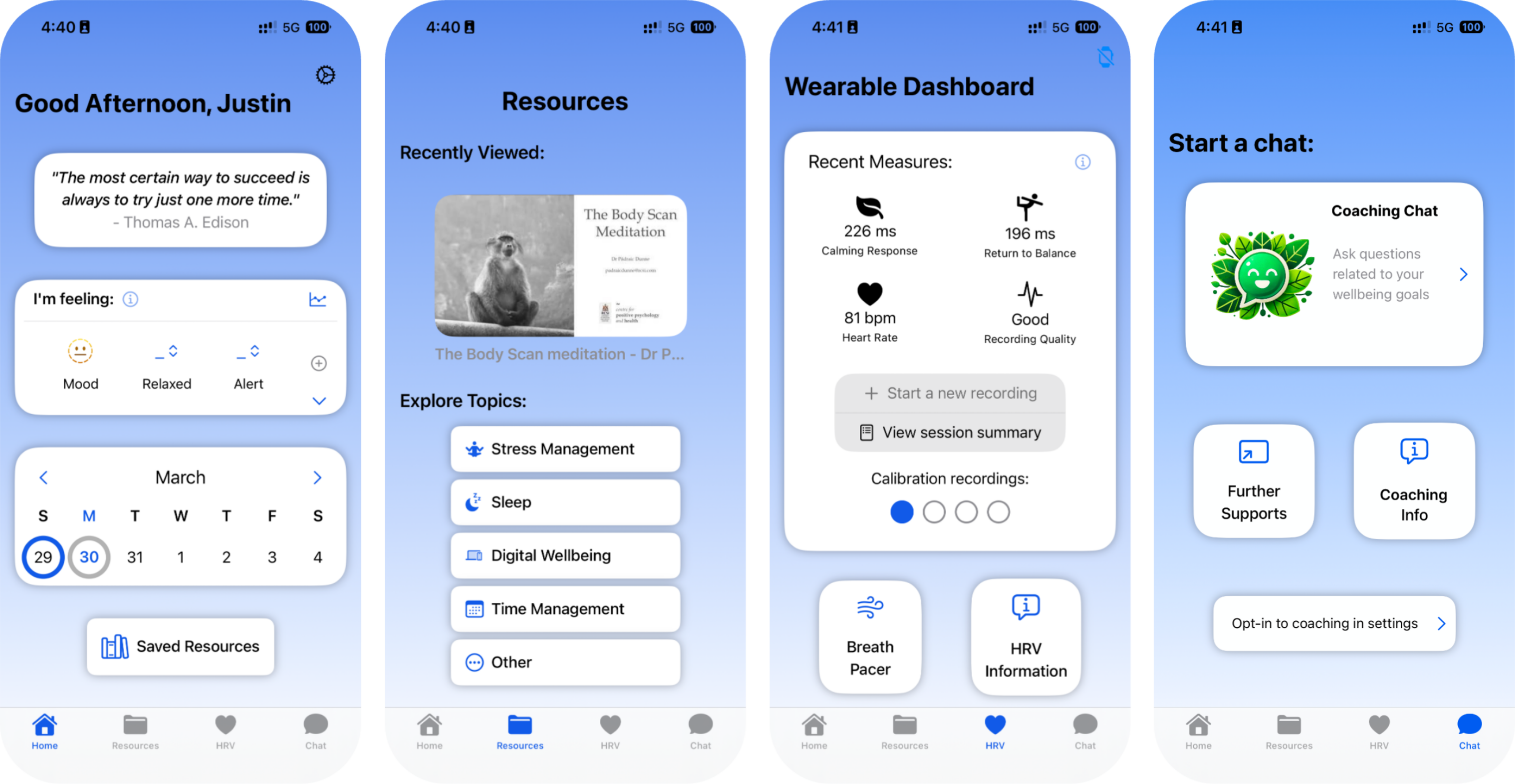
Wellby mobile app tabs showing the home tab (far left), resource tab topics (second to the left), wearable tab (second to the right), and coaching tab (far right).

The co-designed Wellby mobile app was published on the iOS Apple Store and the Google Play Store for students to access. The app was developed by JL, with educational well-being and HRV content curated by JM, PD, and JL. The mobile app includes four tabs (Figure 1): (1) a home tab with student-requested tools, including an optional daily quote, mood check-in and tracker, a clickable calendar with daily to-do lists, and settings where customizable app colors can be chosen; (2) an educational resource tab with evidence-based articles, infographics, and videos on topics requested by students, including stress management, sleep, digital well-being, and time management; (3) a wearable tab that facilitates connection to the Wellby wearable device and offers options to start new recordings, see past recordings, learn about HRV, and engage in breath work; and (4) a health coaching tab that offers optional text-based messaging with certified health coaches around student-requested well-being focus areas. Two certified health coaches were recruited through the Centre for Positive Health Sciences at the Royal College of Surgeons in Ireland (RCSI) and were vetted by RCSI and the participating schools. In future implementations, coaches could be school counselors, health and well-being staff, or community health workers.

**Figure 2.**
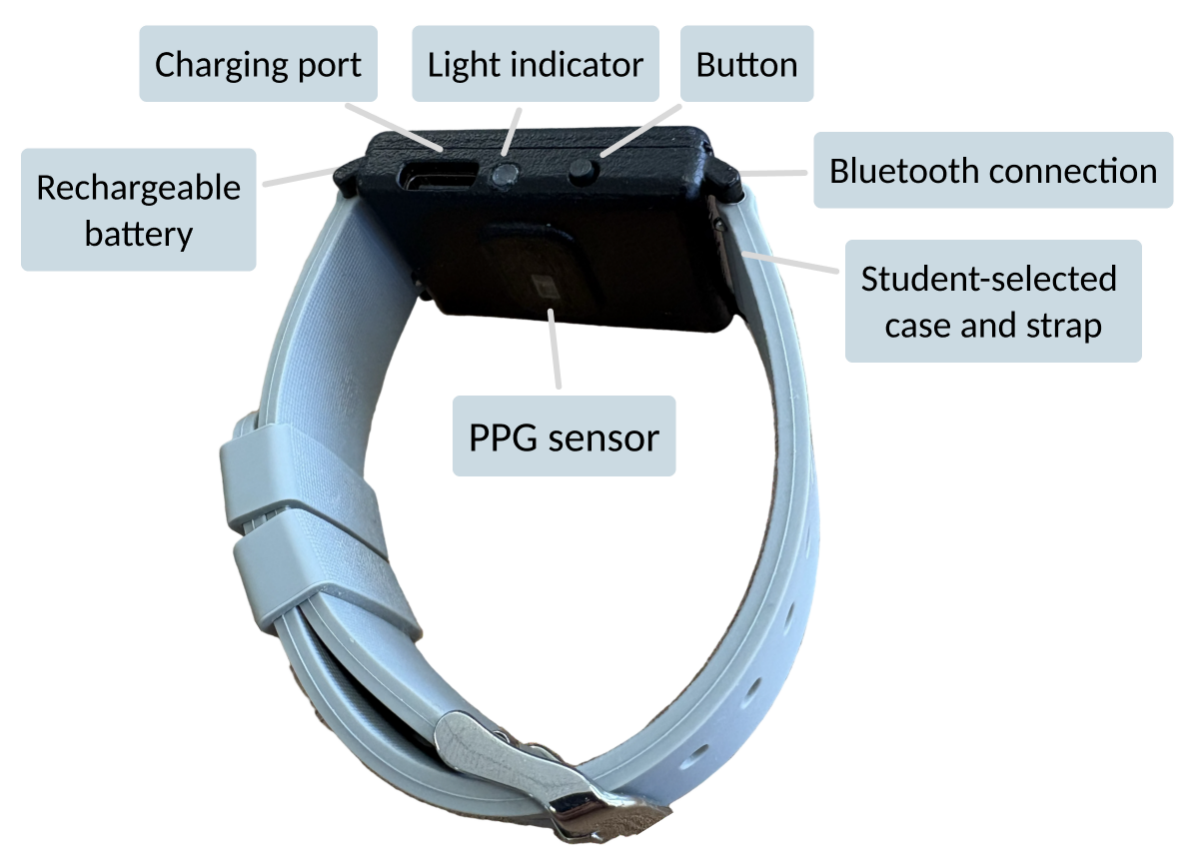
Wellby device feature schematic, including one option for the customizable strap and case colors. PPG: photoplethysmography.

The Wellby wearable is a wrist-worn device that includes a photoplethysmography sensor for tracking heart activity, a rechargeable battery, a Bluetooth connection module, and a light indicator of battery and connection status (Figure 2). This device was designed and assembled by engineering researchers based at RCSI (JL and SJ). Developing a custom device in this study allowed for increased adaptability and customizability of the intervention based on student input throughout the iterative cocreation process. It also enhanced data control, analysis, and security, enabling intentional and transparent data collection and processing. Students indicated in the co-design that the appearance of the device was important to them. Therefore, they were able to request the color of the nylon case, strap design, and strap material for the wearable in this study.

### Study Design

In this study, students tested Wellby for 8 weeks (April-May 2024). The primary outcome measure of acceptability and usability was assessed using the user version of the Mobile App Rating Scale (uMARS) [46], survey questions, qualitative feedback from follow-up focus group discussions, and app engagement measured throughout the study. As secondary outcome measures, well-being questionnaires were collected at baseline. Perceived stress, sleep quality, and overall well-being were measured using the 10-item Perceived Stress Scale (PSS) [47], the Pittsburgh Sleep Quality Index (PSQI) [48], and the EPOCH (engagement, perseverance, optimism, connectedness, and happiness) Measure of Adolescent Well-Being [49], respectively. Well-being questionnaires were only collected at baseline because of students’ limited availability and increased pressure during the final weeks of their school year. We prioritized collecting the primary outcomes for this acceptability and usability study, which was designed to inform a follow-up longitudinal efficacy study with repeated well-being measures. Additional students were recruited at schools A and B who completed the well-being questionnaires without testing Wellby. This group was recruited to compare the well-being of students with and without access to Wellby for potential future efficacy studies on the impact of Wellby on student well-being. No additional students were recruited at school C because all students were involved in testing Wellby.

Students first had access to the Wellby mobile app alone for 4 weeks before receiving the connected wrist-worn HRV monitor for the final 4 weeks of the study. This allowed for the investigation of the acceptability and usability of the mobile app alone and in combination with the wearable device. Four weeks represents a common duration of acceptability studies of wearables for adolescents [33]. The phased approach aligns with the iterative development of cocreated tools [36] and enabled comparison between the intervention components. Students were encouraged to use Wellby as much as they found useful throughout the study period. The uMARS and hour-long focus group sessions were completed at the end of the 8-week study.

Positive design provided the framework to develop the Wellby system [29], and Radical Participatory Design [16] provided the methodological guidelines to promote continuous collaboration with students throughout the cocreation process. As outlined in a previous work [45], students were involved in a needs assessment and co-design sessions to shape the focus and design of Wellby.

Through the study protocol and cocreation approach, we aimed to reduce the potential risks that are presented by digital tools, such as privacy concerns, overuse, and fixation on negative data [20,50]. Data were encrypted in Google Firebase, and access was reserved to approved data controllers (JL and PD). We established an incident reporting protocol for in-app coaches to notify researchers of messages indicating potential violence or harm to students. During participant onboarding, we demonstrated the capabilities of the technology in person and provided multiple avenues for the students to raise questions or concerns during the study, such as through their teacher or an in-app feedback form. To avoid fixation on negative data, evidence-based text and diagrams were provided in the biofeedback tab about the meaning of HRV metrics, and signal quality metrics were included for each recording to enhance transparency about the reliability of the HRV data.

**Figure 3.**
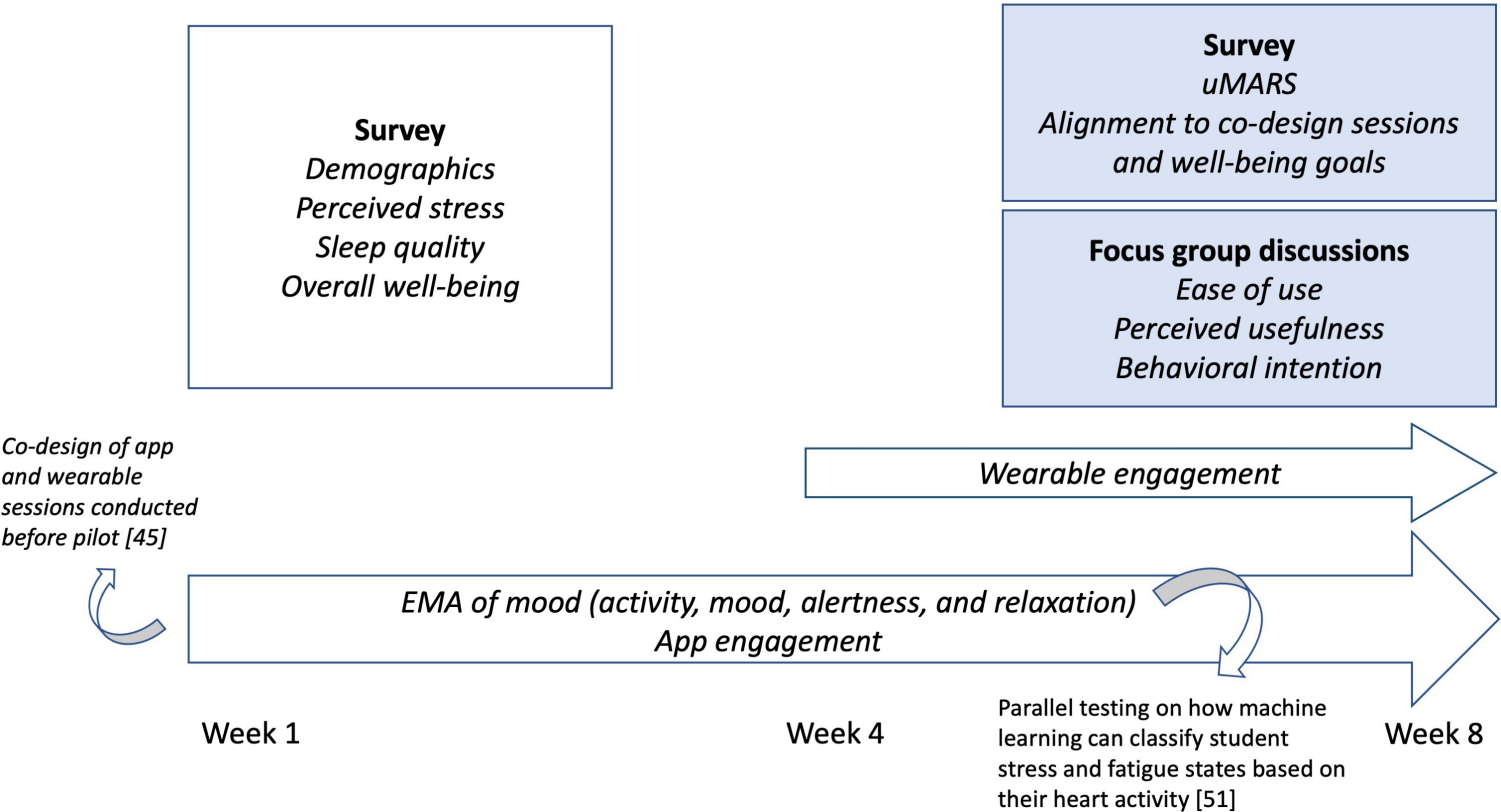
Wellby data collection methods outline of the 8-week study, including pre- and postintervention surveys, continuous data collection, focus group discussions, and connected research papers [45,51]. EMA: ecological momentary assessment; uMARS: user version of the Mobile App Rating Scale.

Additionally, students were encouraged to complete ecological momentary assessments (EMAs) throughout the study and wearable recordings during the final 4 weeks. The EMAs included questions about the student’s current activity, mood (represented as an emoji), and alertness and relaxation level on a scale from 1 to 5. The focus on alertness and relaxation was connected to sleep and stress, previously identified as being the well-being priorities of this cohort [45]. Check-in reminders to engage with the app and complete the EMAs were sent at 3 random times during the school week, provided students had enabled notifications. A separate and parallel study was conducted based on the EMAs and wearable recordings from the testing of Wellby. The objective of this parallel study was to understand how machine learning could potentially be used to classify student stress and fatigue states based on their HRV metrics [51]. Figure 3 illustrates the data collection methods and timepoints throughout this study.

### Questionnaire Measures

The postintervention survey included questions about the study’s alignment with students’ well-being goals and the uMARS questionnaire, providing quantitative data associated with the primary outcome of this study. The uMARS includes 5 subscales and has previously demonstrated an overall internal consistency (Cronbach α) of 0.90 [46]. Similarly, from the responses in our study, the uMARS resulted in good internal consistency (α=0.89). The first 4 subscales of the uMARS focused on engagement, functionality, aesthetics, and information quality, grouped into the “ease of use” of Wellby. The final subscale of the uMARS focused on the perceived impact on awareness, knowledge, attitudes, intention to change, help seeking, and behavior change based on the use of Wellby. Each subscale included questions with responses ranked on a scale from 1 to 5, with 1 being the lowest and 5 being the highest score.

The baseline well-being questionnaires, including the PSS, PSQI, and EPOCH, have been validated for adolescent populations where they demonstrated internal consistencies (Cronbach α) of 0.62, 0.72, and 0.88, respectively [52-54]. Our study demonstrated comparable internal consistency for the PSS (α=0.78), PSQI (α=0.74), and EPOCH (α=0.89). The overall scores for these questionnaires range from 0 to 40 for the PSS, 0‐21 for the PSQI, and 0‐25 for the EPOCH questionnaire, where higher scores indicate greater stress, lower sleep quality, and greater well-being, respectively.

### Data Analysis

To analyze the uMARS results, the first 4 subscales were grouped to evaluate the ease of use of Wellby, while the fifth subscale was used to evaluate the behavioral intention and perceived usefulness in supporting student well-being. Survey questions about the alignment of Wellby with the outcomes of previous co-design sessions and students’ well-being goals were summarized using descriptive statistics.

Qualitative feedback from the focus group discussions was analyzed by two researchers (JL and EB) using reflexive thematic analysis based on Braun and Clarke’s approach [55]. The identified themes were then grouped into ease of use, perceived usefulness, and behavioral intention based on the TAM [42]. Pen profiles were constructed to illustrate these groupings and provide example quotations associated with each subtheme along with an indication of the number of mentions [56]. Focus group participants included students who were able to attend the in-person, postintervention meeting. These took place during the final weeks of the school year. Demographic information of focus group participants was not available for analysis as this was only collected in the onboarding survey.

App engagement was recorded on Google Firebase, which saved data to indicate the app tabs and features that the students interacted with throughout the study. The engagement data and well-being metrics were summarized across each school using descriptive statistics.

## Results

### Student Well-Being Assessments

The well-being questionnaires administered as part of the onboarding survey are summarized based on demographic categories in Table 1. Notably, the results demonstrate a higher perceived stress for students at school B, the all-female school, and for female participants overall. Similarly, perceived sleep quality was lowest at school B and for female students. The EPOCH assessment of overall adolescent well-being indicated the highest well-being in students at school A and in male students (Table 2).

**Table 2.** Student well-being questionnaire results by demographic for the Perceived Stress Scale (PSS), the Pittsburgh Sleep Quality Index (PSQI), and the EPOCH^a^ Measure of Adolescent Well-Being.

Category	PSS, mean (SD)	PSQI, mean (SD)	EPOCH, mean (SD)
School			
School A (n=16)	18.5 (4.0)	6.8 (2.3)	16.7 (3.1)
School B (n=11)	24.2 (5.6)	9.5 (4.0)	15.3 (3.2)
School C (n=16)	19.8 (7.8)	8.1 (3.6)	15.1 (4.0)
Age (years)			
16 (n=12)	16.5 (7.4)	7.5 (4.3)	15.9 (4.9)
17 (n=25)	21.2 (4.9)	7.7 (3.0)	15.7 (2.9)
18 (n=3)	25.3 (7.6)	10.0 (3.6)	17.8 (1.0)
19 (n=3)	25.0 (5.6)	9.7 (1.5)	13.7 (2.3)
Gender			
Female (n=27)	23.1 (5.5)	8.7 (3.3)	15.0 (2.7)
Male (n=13)	15.7 (5.6)	5.9 (2.9)	18.0 (2.9)
Nonbinary (n=1)	16 (N/A^b^)	13 (N/A)	20 (N/A)
Prefer not to say (n=2)	18.0 (1.4)	8.5 (0.7)	10.1 (7.3)
Race and ethnicity			
Asian (n=3)	26.7 (8.4)	13.3 (3.8)	10.8 (5.2)
Black or Black Irish (n=2)	25.0 (12.7)	12.0 (1.4)	18.4 (2.3)
White/other European (n=7)	17.7 (3.5)	6.6 (2.9)	16.3 (4.1)
White African (n=1)	19 (N/A)	8 (N/A)	15.3 (N/A)
White Irish (n=30)	20.2 (6.1)	7.5 (3.0)	16.0 (3.0)
Total (N=43)	20.4 (6.3)	8.0 (3.4)	15.8 (3.5)

a EPOCH: engagement, perseverance, optimism, connectedness, and happiness.

b N/A: not available.

Because of the notable gender differences in the well-being questionnaires and the potential confounding effect of the all-female school, results were analyzed by gender within each school. To increase sample size and generalizability, data from the comparison group (who did not test Wellby) were combined with the intervention group for this gender-based analysis.

Only data from female and male students are presented in Table 3 because these groups had sample sizes that enabled the use of the Mann-Whitney *U* tests for schools A and C to compare results from female and male students. School B was excluded from statistical testing due to the absence of male participants. For school A, Mann-Whitney *U* tests revealed no significant gender differences for PSS scores (*U*=79.5, *P*=.90, *r*=0.031), PSQI scores (*U*=77.5, *P*=.82, *r*=0.051), or EPOCH total scores (*U*=77.5, *P*=.82, *r*=0.051). At school C, a significant gender difference was found for PSS scores, with females reporting higher stress than males (*U*=0, *P*=.007, *r*=0.770). While effect sizes suggested greater sleep quality in males, no significant differences were found for PSQI scores (*U*=6, *P*=.08, *r*=0.514) or EPOCH scores (*U*=9, *P*=.19, *r*=0.385).

Students’ well-being goals set at the beginning of the study included elements of lifestyle medicine (daily activity, eating well, relationships, sleep, and stress management) and digital well-being, identified by students as relevant well-being needs in the previous co-design sessions. Sleep and stress management were the most chosen categories for the total student selections (Table S1 in Multimedia Appendix 1). Daily activity and eating well were also frequently chosen, particularly at schools A and C.

**Table 3.** Student well-being questionnaire results based on gender at each school.

School^a^	PSS^b^, mean (SD)		PSQI^c^, mean (SD)		EPOCH^d^, mean (SD)	
	Female	Male	Female	Male	Female	Male
School A (n=15/11)	19.3 (4.4)	19.2 (3.4)	6.8 (2.0)	7.5 (3.0)	17.3 (3.9)	17.4 (2.7)
School B (n=16/0)	23.8 (6.1)	N/A^e^	9.5 (4.1)	N/A	15.2 (2.9)	N/A
School C (n=9/5)	24.8 (4.4)	15.3 (3.0)	9.3 (2.5)	5.5 (3.0)	14.3 (1.9)	16.7 (3.1)
Total (n=40/15)	22.4 (5.4)	18.1 (3.6)	8.3 (3.1)	6.9 (3.1)	15.9 (3.4)	17.2 (2.7)

a Participant numbers presented as n=female/male.

b PSS: Perceived Stress Scale.

c PSQI: Pittsburgh Sleep Quality Index.

d EPOCH: EPOCH Measure of Adolescent Well-Being, which assesses 5 positive psychological characteristics (engagement, perseverance, optimism, connectedness, and happiness).

e N/A: not applicable, all-female school.

### Survey Data

#### Survey Overview

At the end of the 8-week study, students (n=29) completed the postintervention survey, aligning with the final weeks of their school year, which may have contributed to the 67% (29/43) response rate.

#### Ease of Use

On the first 4 uMARS subscales, the mean of the total scores was 4.1 (SD 0.7). School B had the highest average score for each subscale, 4.0 (SD 0.9) for engagement and 4.4 for functionality (SD 0.3), aesthetics (SD 0.8), and information quality (SD 0.7), compared to the other schools. Based on the student responses grouped across all schools, information quality had the highest overall score of 4.2 (SD 0.6), while engagement had the lowest overall score of 3.8 (SD 0.7). The functionality, aesthetic, and information quality subscales all reported average scores greater than 4.0 for the grouped responses (Table 4).

**Table 4.** The uMARS^a^ “ease of use” subscale scores by school site^b^.

uMARS subscale	School A (n=6)	School B (n=7)	School C (n=16)	Total (n=29)
Engagement	4.0 (0.7)	4.0 (0.9)	3.7 (0.7)	3.8 (0.7)
Functionality	4.3 (0.4)	4.4 (0.3)	3.9 (0.6)	4.1 (0.6)
Aesthetic	3.8 (0.9)	4.4 (0.8)	4.2 (0.8)	4.1 (0.8)
Information quality	4.2 (0.4)	4.4 (0.7)	4.2 (0.7)	4.2 (0.6)

a uMARS: user version of the Mobile App Rating Scale.

b Data are presented as mean (SD).

#### Perceived Usefulness and Behavioral Intention

On the final uMARS subscale, the perceived usefulness of Wellby was indicated by the highest scores of 3.9 (0.8) and 3.8 (1.0) for increased help seeking and well-being awareness, respectively (Table 5). Regarding behavioral intention, uMARS results indicated moderate levels of intention to change (mean 3.7, SD 1.0) and anticipated behavior change (mean 3.6, SD 1.2) because of Wellby.

Of the student respondents who indicated prior participation in the co-design sessions for Wellby (n=24), 88% (21/24) and 83% (20/24) felt that the wearable and the app, respectively, aligned “extremely well” or “somewhat well” with their feedback from co-design sessions. Remaining responses indicated students felt “neutral” about the alignment of Wellby with the co-design session outcomes. Responses from all students, including those not involved in the co-design sessions, indicated that a majority (22/29, 76%) felt Wellby aligned “extremely well” or “somewhat well” with their well-being needs (Table S2 in Multimedia Appendix 1).

**Table 5.** The uMARS^a^ perceived impact scores at each school site^b^.

uMARS perceived impact questions	School A (n=6)	School B (n=7)	School C (n=16)	Total (n=29)
Awareness: Wellby has increased my awareness of the importance of addressing aspects of my well-being	3.5 (1.0)	3.9 (1.3)	3.9 (0.8)	3.8 (1.0)
Knowledge: Wellby has increased my understanding of my well-being	3.2 (1.0)	3.6 (1.1)	3.8 (0.9)	3.6 (1.0)
Attitudes: Wellby has changed my attitudes toward improving my well-being	3.2 (1.6)	3.6 (1.1)	3.9 (0.9)	3.7 (1.1)
Intention to change: Wellby has increased my motivation to address certain aspects of my well-being	3.7 (0.8)	3.6 (1.5)	3.9 (0.9)	3.7 (1.0)
Help seeking: Wellby would encourage me to seek further well-being support (if I needed it)	4.2 (0.8)	3.6 (0.8)	3.9 (0.8)	3.9 (0.8)
Behavior change: Use of Wellby will increase aspects of my well-being	3.0 (0.9)	3.9 (1.3)	3.6 (1.2)	3.6 (1.2)

a uMARS: user version of the Mobile App Rating Scale.

b Data are presented as mean (SD).

### Focus Group Feedback

#### Focus Group Overview

Structured focus groups were conducted in person at the end of the intervention with students at school A (n=6) and school B (n=5). Due to time constraints at school C, less structured feedback from students was gathered, which is summarized under the “ease of use” section. Themes from the discussion were grouped based on TAM categories, and subthemes are detailed using the number of mentions for each theme, a student quotation, and a school indicator for each quote.

#### Ease of Use

**Figure 4.**
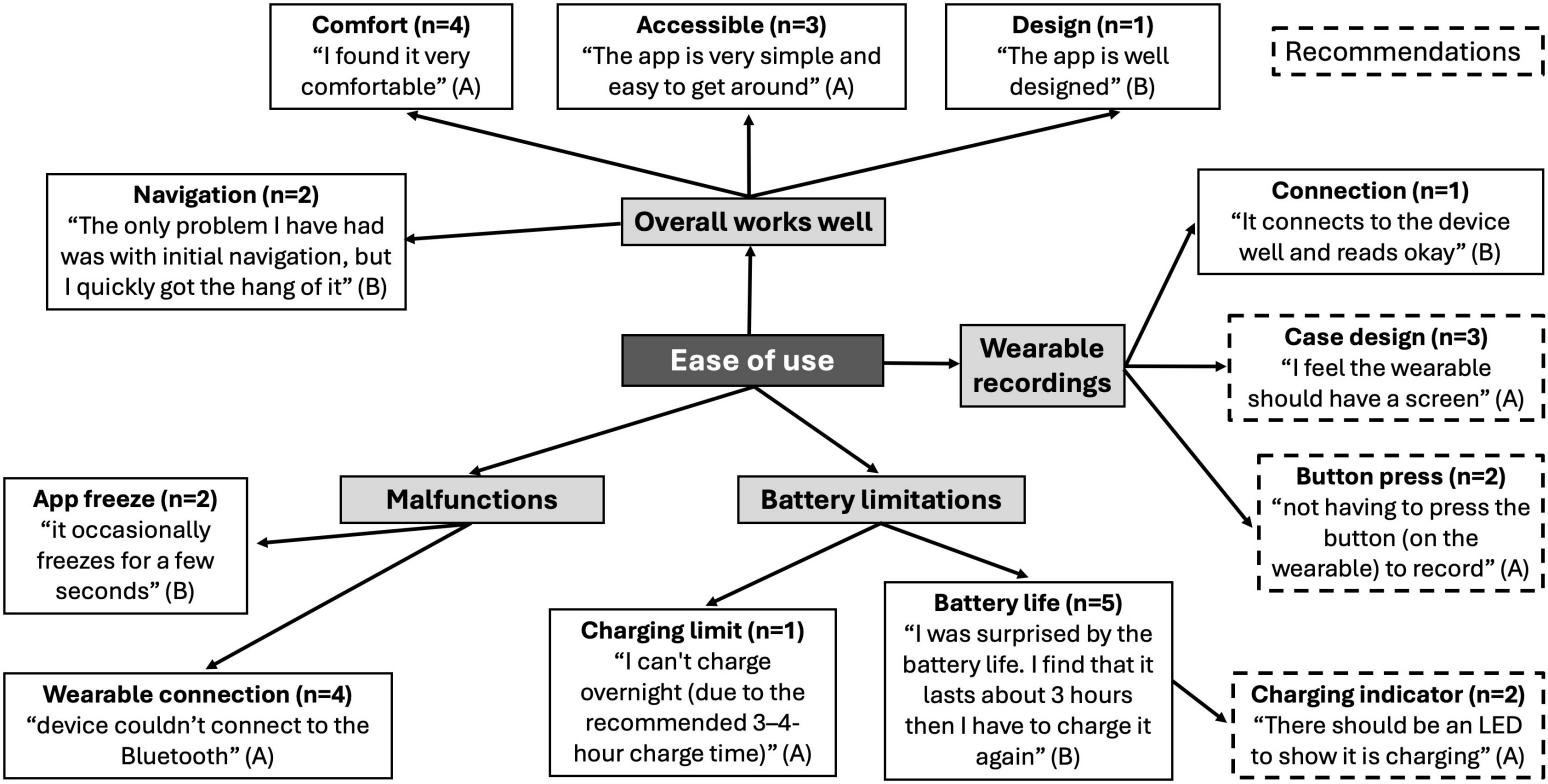
Student focus group feedback on the “ease of use” of Wellby grouped into major themes. The subthemes include the frequency (n), an example quote, and the school associated with each student quote (represented by A, B, or C). Subthemes that were recommendations from students are indicated with dashed borders.

There was an overall positive response to the ease of use of Wellby, with many students noting that the wearable was comfortable and that the app was “easy to get around” (school A) (Figure 4). Some students noted that it took time to initially understand the app navigation. While one student noted that the app “connects to the device well” (school B), others (n=4) noted challenges with Bluetooth connection, particularly on the Android version of the mobile app. Other limitations included occasional app freezing and battery life. Several students noted that the battery life was not long enough (n=5), and one student specified that they would like to charge it overnight but were unable to due to the recommended 3‐4 hour charging time. Students also recommended ways to improve the ease of use of Wellby by including a light indicator for different charging stages on the wearable and making it possible to start a heart activity recording without having to press the button on the wearable. Additionally, some students (n=3) noted that they would like something on the face of the wearable, such as a screen or clock. Feedback from school C indicated that students didn’t see the wearable as socially acceptable with their school’s fashion norms. They recommended changes including making the device rounder, having a shiny or metallic material, and including more color options.

#### Perceived Usefulness

**Figure 5.**
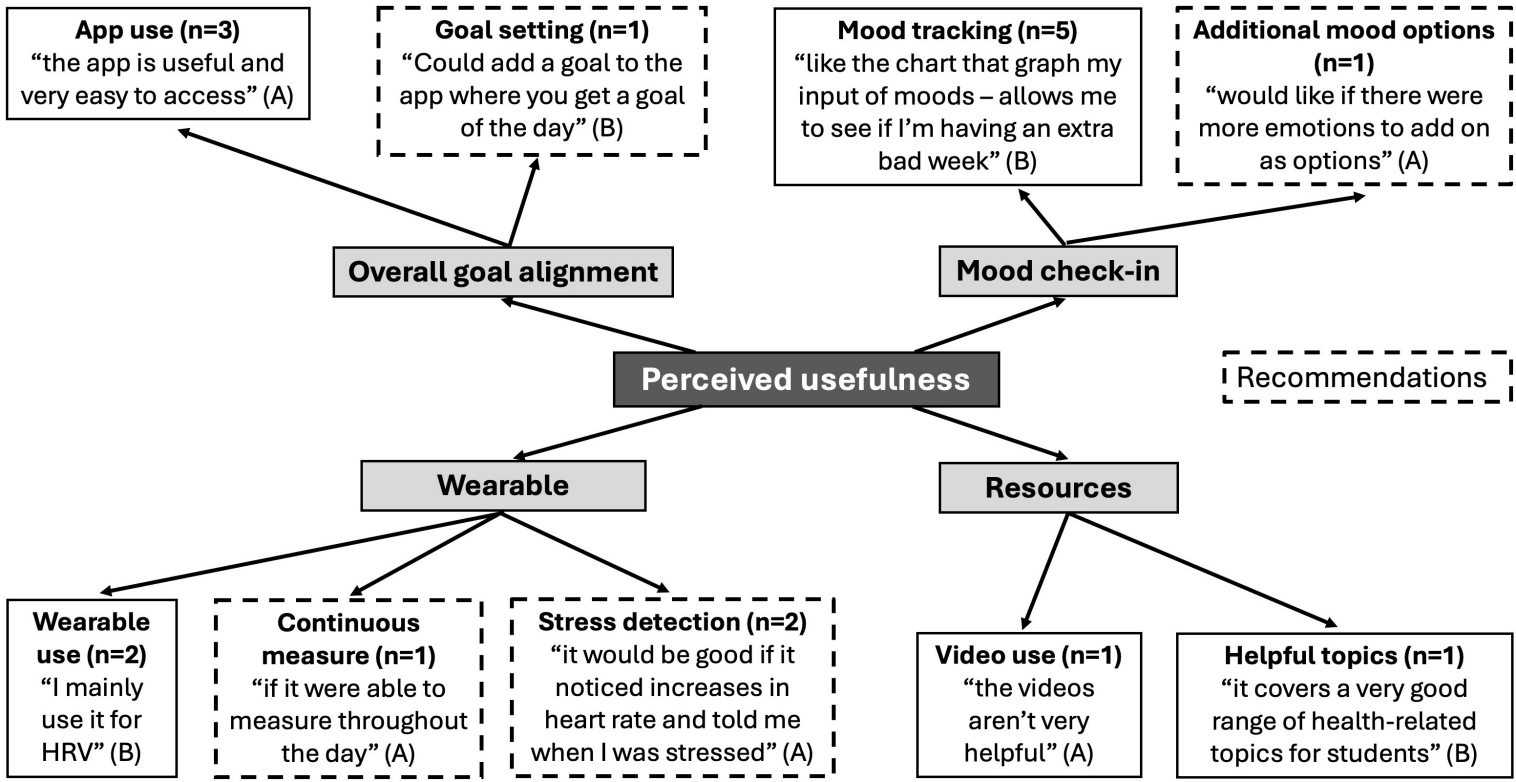
Student focus group feedback on the “perceived usefulness” of Wellby grouped into major themes. The subthemes include the frequency (n), an example quote, and the school associated with each student quote (represented by A, B, or C). Subthemes that were recommendations from students are indicated with dashed borders.

Students indicated an overall alignment of Wellby with their well-being goals (Figure 5). One student recommended adding a feature to the app to specify and track student goals. The most mentioned app feature was the mood tracker, one student sharing that it “allows [them] to see if [they’re] having an extra bad week” (school B) and another that they “like the check-in feature” and “spend ages looking through the emojis to choose [their] mood” (school B). To further enhance the mood check-in, a student recommended adding more options to this feature so that they can input more details about their current moods.

Students had mixed opinions about the usefulness of app resources, with one student noting that they “cover a very good range of health-related topics for students” (school B), while another noted that the video resources “aren’t very helpful” (school A).

Students also noted feedback about the wearable, one describing that they mainly use Wellby to track their HRV. They also noted that they would like for the wearable to be able to monitor their heart activity continuously throughout the day and notify them of potential increases in stress levels.

#### Behavioral Intention

**Figure 6.**
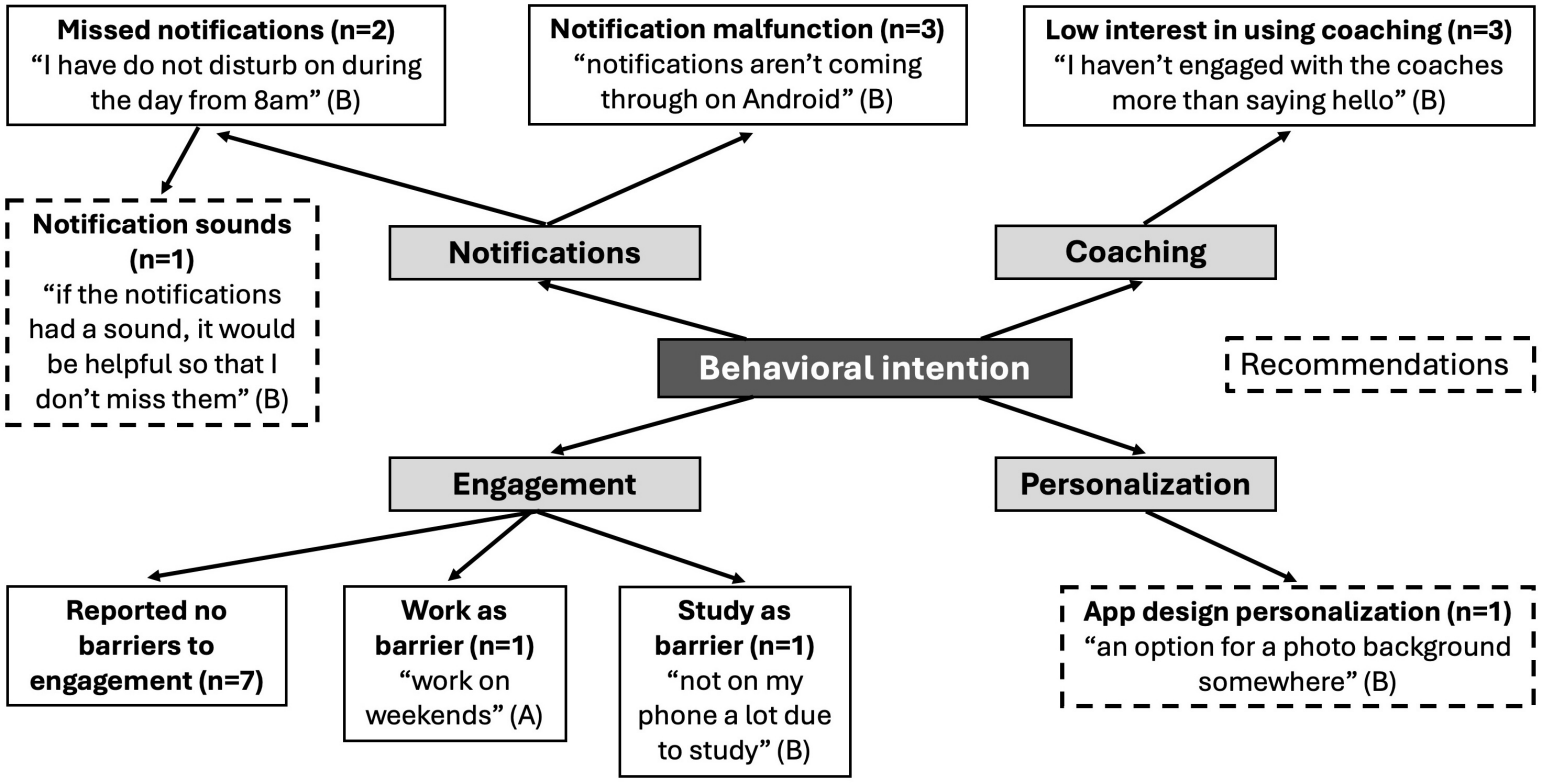
Student focus group feedback on the “behavioral intention” of Wellby grouped into major themes. The subthemes include the frequency (n), an example quote, and the school associated with each student quote (represented by A, B, or C). Subthemes that were recommendations from students are indicated with dashed borders.

Student feedback from the focus group also included barriers and facilitators of students’ engagement with Wellby. Several students (n=3) noted that they had little interest in messaging the health coach provided through the app (Figure 6). Other students missed notifications due to focus features on their phones or malfunctions on Android. One student recommended adding sounds to notifications so that they remember to engage with the app. When asked if there were external factors preventing them from using Wellby, one student indicated that they had work on the weekends, another noted their focus on studying, while most (n=7) indicated no external barriers to engagement. Students also commented on the benefits of personalizing Wellby, such as the app background color or wearable strap material. One student recommended enhanced personalization through choosing custom background images for the app.

### Wellby Engagement Metrics

**Figure 7.**
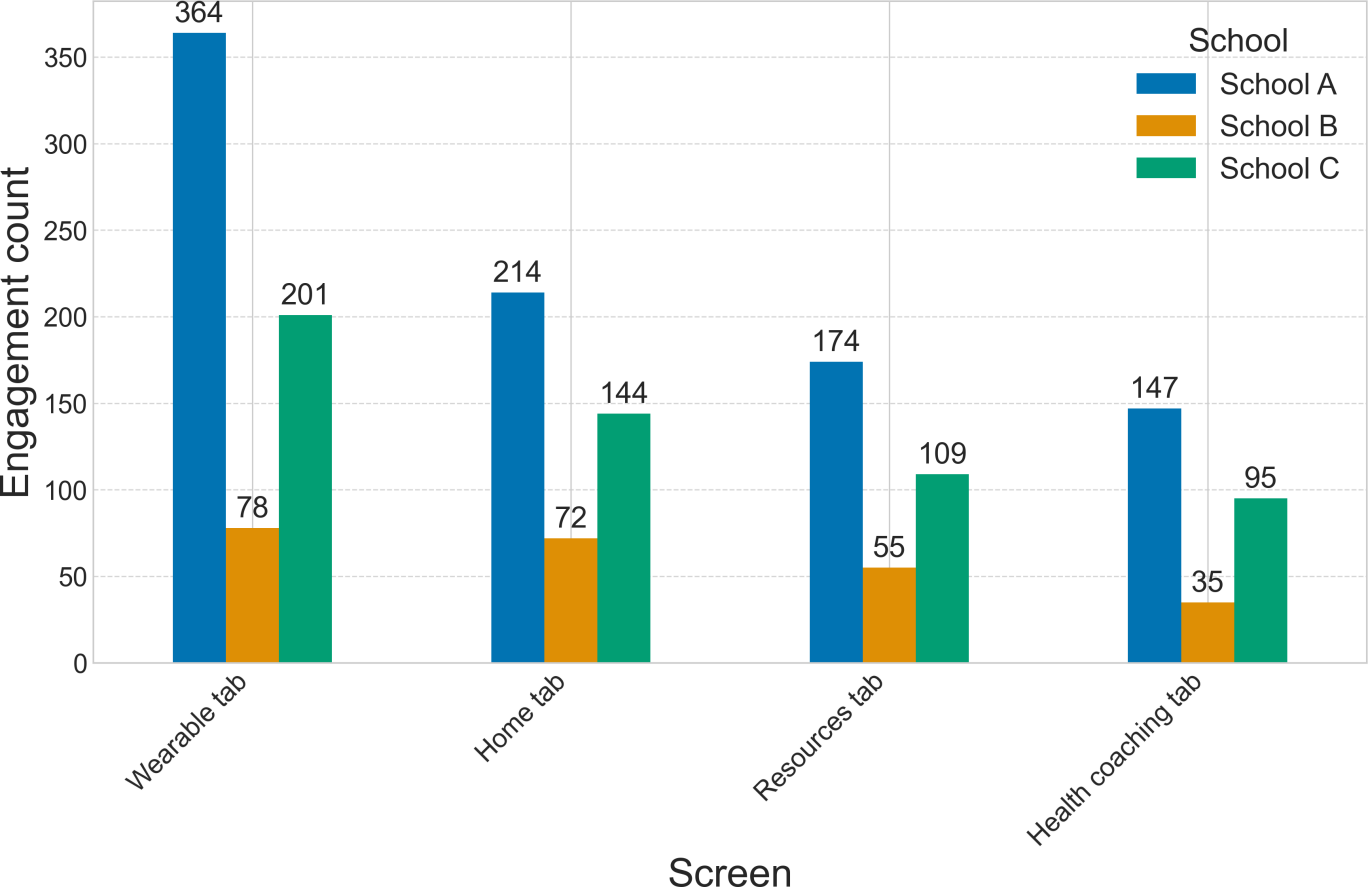
Student engagement counts with the Wellby app represented across each school and grouped by app tab.

App engagement data throughout the 8 weeks was summarized based on the total engagement with the 4 app tabs, separated by school, and the overall engagement with each app feature. The wearable tab had the highest engagement, followed by the home tab, resource tab, and coaching tab, for each school. Engagement was highest at schools A and C, which had 5 more students involved in the testing than school B (Figure 7). The app features that allowed students to customize the background color on the app had the highest engagement, with 417 total clicks throughout the study period. The following 4 features with the most engagement included completing mood check-ins, viewing specific resource topics, recording HRV, and viewing HRV summary (Table S3 in Multimedia Appendix 1).

**Figure 8.**
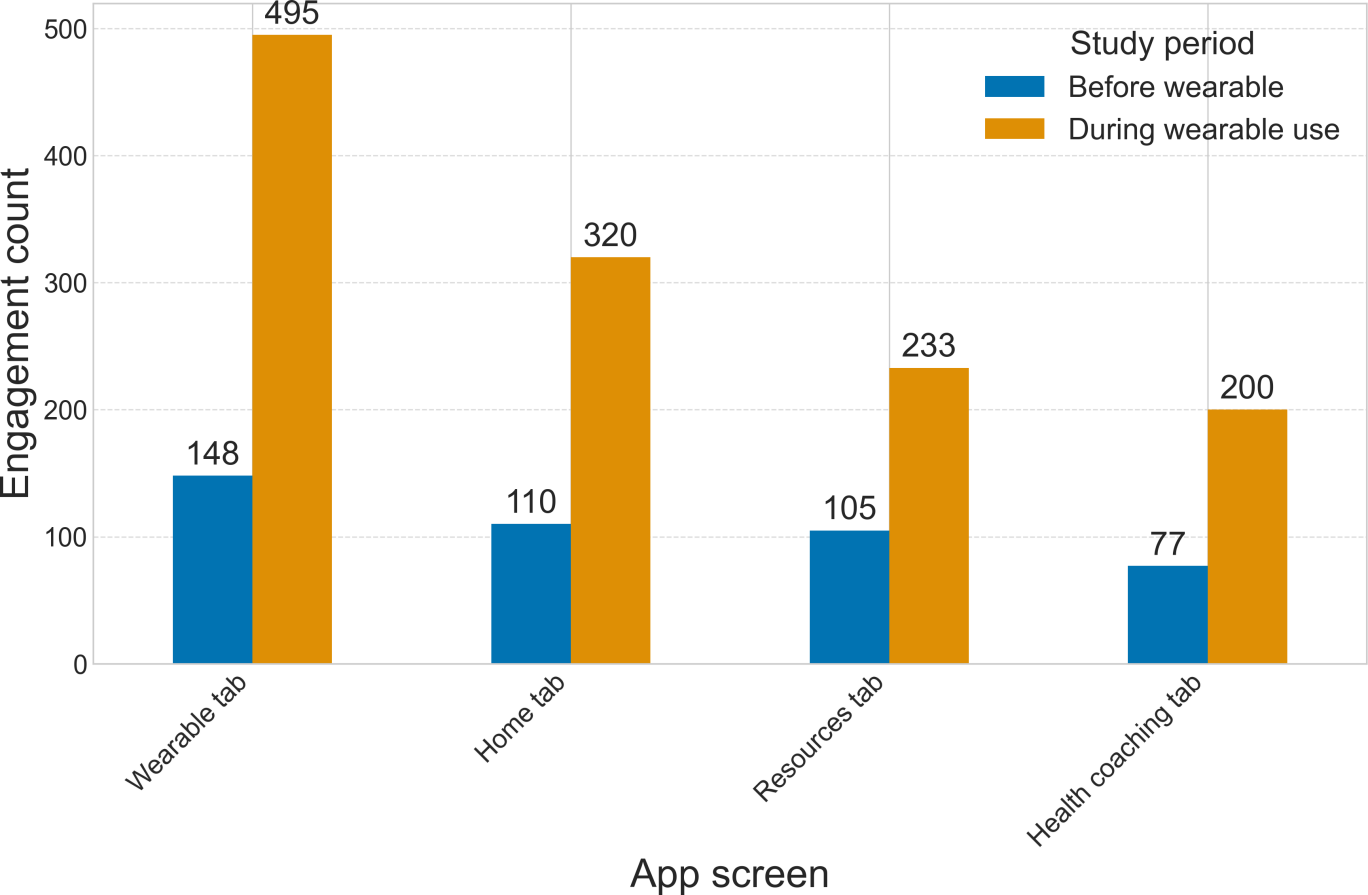
Student engagement counts with the Wellby app tabs during the first 4 weeks of the study (before wearable access) compared to the final 4 weeks (when students had access to the wearable device).

Since students had access to the mobile app alone for the first 4 weeks of the study before being given wearables, the engagement was analyzed during both time periods. Student engagement with each app tab increased substantially, ranging from 123 to 347 additional interactions per tab, during the final 4 weeks of the study when they had access to the wearable device (Figure 8). The wearable tab included access to HRV data summaries, a wearable connection to begin a new recording, information on interpreting HRV, and a customizable breath pacer.

## Discussion

### Principal Findings

This mixed methods acceptability and usability study demonstrated the successful alignment of the co-designed Wellby intervention with the needs and preferences of Irish secondary school students. Additionally, secondary baseline well-being measures captured student well-being needs.

The identification of stress and sleep as the most selected well-being goals aligned with the baseline questionnaire results that indicated high stress and low sleep quality in student participants. The mean overall PSS score (mean 20.4, SD 6.3) was substantially higher than scores reported from a similar cohort in Greece (mean 12.6, SD 8.1) [57], while the mean overall PSQI score (mean 8.0, SD 3.4) was above the 6.0 cutoff for poor sleep in young adults [58]. Additionally, the results of the well-being questionnaires varied across demographics and schools, with the highest perceived stress and lowest sleep quality in female students and at the all-female school. This is consistent with stress-related gender differences identified in similar studies of adolescents using digital health tools [59]. Our findings highlight the context-specific well-being needs that exist both between and within different school environments, echoing the results of the needs assessment and co-design phase of Wellby [45]. The gender differences observed from the well-being questionnaires at school C may indicate that the needs of Youthreach students when they join the program differ between genders. Future research could further examine the well-being and acceptability differences among demographics, including potential gender-specific differences, and how personalized features can enhance the adaptability of digital tools to meet a range of student needs.

The high uMARS scores and strong alignment with students’ goals likely reflect Wellby’s close adherence to student input during co-design. The overall average score for the first 4 subscales in the uMARS (mean 4.1, SD 0.7) is comparable to the uMARS evaluations of the highest rated available mental health apps (mean 4.1‐4.3) assessed in a review by Ko and Woo [60], compared to those with the lowest ratings (mean 2.9‐3.3) out of the 41 eligible apps reviewed on the same 4 subscales. The results also revealed that the alignment of Wellby with students’ goals was higher for those in the co-design sessions (21/24, 88%) compared to students overall (22/29, 76%). Additionally, students shared recommendations for adjusting the wearable appearance to match their style and for additional features, such as goal tracking. These requested improvements to Wellby may explain why the alignment responses were not higher. Overall, these findings add to existing evidence on the effectiveness of participatory design processes in producing tools that are appropriate and acceptable for adolescents [17].

Barriers to engagement that emerged in the focus groups included technical challenges and environmental constraints such as schoolwork and nonacademic commitments. While technological issues are common barriers in mobile health research, there is a need to more thoroughly examine the system-level influences on students’ engagement with digital well-being tools [61]. Researchers might investigate these by working with students’ guardians, teachers, and school staff members to gain a wider perspective on the implications and considerations for implementing digital well-being supports in an educational context. Together they could explore how to integrate these tools into schools’ existing support pathways for students such as through school counselors, physical education, or health teachers. The financing for these supports could be allocated through governmental budgets for school well-being resources, which schools can then allocate to the most appropriate resources for their students. For example, the national health service in Ireland provides free digital cognitive behavioral therapy through various referral pathways, and Harty et al [62] demonstrated how this enables digital well-being support tools to be distributed at scale.

The recurring themes related to challenges with the study technology also have important implications for participant engagement. Technological barriers noted by students, such as the short battery life of the wearable and Android compatibility errors, likely reduced sustained engagement. This was underscored by the engagement subscale having the lowest “ease of use” score on the uMARS. Several feedback items from students were also recommendations for features that could increase the interactivity and student interest in Wellby, such as goal trackers, updates to the mood tracking and wearable feedback, and increased options for personalization.

Students additionally expressed their enjoyment in customizing the wearable case and strap, potentially increasing feelings of ownership and trust. While this study aimed to foster collaboration with students, there are several ethical considerations when working with adolescents [30], and technical issues can reduce trust in these types of digital interventions [63]. Notably, previous research demonstrated that trust in technology is a crucial driver of acceptability, particularly for adolescents [64]. Therefore, potential technical issues should be identified early, for example, through short-term tests with students before longer interventions. Future research could further investigate how to promote trust among participants through selected features and collaborative approaches to these interventions.

Student engagement patterns with Wellby indicated that app color personalization, mood check-ins, viewing educational resources, and HRV recordings were the most common features used during this study. This agrees with qualitative feedback indicating that students were most interested in personalized features, the mood check-in, and the wearable component of Wellby. Despite conflicting opinions on the usefulness of the in-app resources in focus groups, the information quality achieved the highest score (mean 4.2, SD 0.6) out of the uMARS subscales. Along with students’ frequent engagement with the educational resources, this high uMARS score suggests that several students found the evidence-based well-being content to be relevant and beneficial to them.

After students received wearable devices at the study midpoint, engagement increased across all Wellby app tabs. Although technical challenges with the wearable were reported in focus group discussions, students emphasized their interest in the wearable and in features for future iterations. The engagement increases following wearable implementation, alongside qualitative feedback and moderate uMARS scores for intention to change and behavior change, suggests that the devices were a main driver of student engagement and demonstrate the potential of this tool to encourage behavior change in adolescents. Rather than providing an additional mobile app alone, wearables offer a tangible representation and reminder of students’ commitment to enhancing their well-being. Wearable devices may provide valuable tools to catalyze student behavior change and the establishment of habits that can support them as they grow older, particularly by providing a device that can bridge the digital and physical sides of many adolescents’ experiences.

Student engagement patterns and feedback also indicated students’ overall interest in self-tracking by recording their heart activity and mood over time. This suggests that adolescents may prefer self-directed wellness approaches over guided interventions like coaching or prescriptive content, potentially connecting to their developmental needs for greater autonomy and identity formation. As self-tracking tools become more widely accessible and used, there is a need to provide targeted and mindful technologies that offer tools for adolescents’ inclination toward self-exploration while avoiding the potential for excessive self-monitoring or overreliance on digital tools [65].

Students provided recommendations for the iterative development of Wellby, including increased stress-tracking features, mood-tracking options, and customizable features. This aligns with a main recommendation from a similar co-design study of European adolescents that emphasized the importance of a personalized interface [36]. Social acceptability is another important consideration for this cohort, which emerged in this study, and must be considered for adolescent-centered technologies, particularly wearable technologies that are highly visible. Understanding the psychosocial impacts of technologies can be aided using scales such as the Wearable Acceptability Scale, which offer ways to measure the social acceptability of wearables [66]. Mindful feature design and evaluation helped students to gain an increased sense of ownership and autonomy when using the Wellby system, aligning with the overall goal of cocreating Wellby with students.

### Limitations

One limitation of this study is the short duration. A longer study with follow-up assessments could identify any novelty effects associated with the introduction of a wearable device and capture sustained engagement and actions resulting from students’ behavioral intention beyond 8 weeks. Additionally, the custom wearable device experienced technical difficulties, including battery limitations, Bluetooth connectivity issues, and Android-specific bugs, which may have influenced students’ perceptions of acceptability. These highlight the challenge of developing custom technologies for adolescents and managing students’ expectations. The sample size, while meeting recommended guidelines for usability studies, was relatively small with 43 participants across 3 schools, limiting the generalizability of findings to broader adolescent populations and the analysis of subgroup differences. The postintervention survey response rate of 67% (29/43) and the small focus group sizes should also be considered when interpreting findings, as this may introduce response bias toward students who were more engaged with the study. Future studies should consider timing intervention endpoints when students are more likely to engage by collaborating with school staff to avoid busy academic periods.

The substantial overlap of students involved in both the co-design sessions and intervention testing (38/43, 88% of participants) presents another potential limitation and methodological consideration. This overlap may have introduced familiarity bias due to students’ prior interest and knowledge in the intervention, which could have inflated the acceptability scores. However, this also enabled assessment of participatory fidelity by gathering feedback on whether the intervention reflected students’ input from the co-design session and revealed higher alignment of Wellby with the well-being goals of this cohort compared to students not involved in co-design sessions. When designing future interventions that include participatory design methods, researchers should consider the impacts of prior participatory engagement on the intervention outcomes. Involving the same participants throughout may be appropriate for cocreation studies focused on iterative design and fidelity [67]. However, for studies focused on the generalizability of intervention efficacy and usability, researchers could consider separating participants into cohorts based on prior engagement to control for familiarity bias and enable comparison of acceptability and usability outcomes between these groups.

Future studies should aim to capture a diverse adolescent population by recruiting from different geographic and socioeconomic areas. For example, in Ireland, this could include schools identified by the Delivering Equality of Opportunity in Schools program in both rural and urban areas. Additionally, researchers should collect comprehensive demographic data, such as socioeconomic indicators, to contextualize the outcomes and promote generalizability and reproducibility of their findings. Finally, as this study was conducted in the Irish secondary school context, the findings may not be directly transferable to other cultural or educational environments without consideration of local contexts and adolescent well-being needs.

### Conclusions

This acceptability and usability study provides evidence that co-designed digital well-being tools can achieve high levels of user acceptance among Irish secondary school students when developed through participatory processes. The strong alignment between Wellby and students’ well-being goals, as shown by both quantitative uMARS scores and qualitative feedback, supports the value of involving adolescents as equal partners throughout the design and evaluation process. Students particularly valued self-tracking features including mood tracking and HRV monitoring features, while they engaged less with educational resources and text-based coaching. This was underscored by the considerable increases in app engagement following the introduction of the wearable. This reflects the importance of creating adolescent-centered tools that consider developmental needs such as increased autonomy and identity formation in this age group. Despite some technical limitations, the TAM framework effectively captured students’ experiences and highlighted the importance of ease of use, perceived usefulness, and behavioral intention in capturing the acceptance and usability of digital health interventions.

As adolescents increasingly avail themselves of technologies to aid in self-expression, identity formation, and habit building, these technologies become intertwined with their well-being journeys. Consequently, the design choices and features offered by these technologies influence the ability of adolescents to pursue their unique well-being goals and navigate challenges throughout adolescence. Participatory engagement of relevant stakeholders, well-being–informed design, and iterative testing can help to ensure that new technologies provide adolescents with appropriate support. In exploring the acceptability and usability of Wellby, this study highlighted the value that young people have for personalizable and aesthetic technology, features for self-exploration, and the ability to develop a sense of ownership and autonomy through these tools.

The presented findings provide insights for developers and researchers working to create adolescent-centered digital well-being tools. In addition to actionable student insights for Wellby and similar technologies, this mixed methods study contributes to the growing evidence base supporting participatory design methodologies in creating relevant technologies that can help adolescents to thrive.

## Supplementary material

10.2196/79381 Multimedia Appendix 1 Additional data tables outlining student well-being goals, survey responses, and app engagement counts.

## Abbreviations

EMA: ecological momentary assessment
EPOCH: engagement, perseverance, optimism, connectedness, and happiness
HRV: heart rate variability
PSQI: Pittsburgh Sleep Quality Index
PSS: Perceived Stress Scale
RCSI: Royal College of Surgeons in Ireland
TAM: technology acceptance model
TFA: theoretical framework of acceptability
uMARS: user version of the Mobile App Rating Scale
UTAUT: unified theory of acceptance and use of technology
