# Supplementary material for: Evaluation of Wellby, a Cocreated Mobile App and Wearable to Support Stress Management and Overall Well-Being: Mixed Methods Acceptability and Usability Study
Source: JMIR Hum Factors. 2026 Apr 9;13:e79381. doi: 10.2196/79381 (PMC13064969; doi:10.2196/79381)
Supplement: Multimedia Appendix 1 [file humanfactors-v13-e79381-s001.docx]

**Table S1.** The well-being goals selected by students at each school.

| Category | School A | School B | School C | Total |
| --- | --- | --- | --- | --- |
| Sleep | 9 | 9 | 9 | 27 |
| Stress management | 10 | 6 | 9 | 25 |
| Daily activity | 11 | 4 | 6 | 21 |
| Eating well | 9 | 3 | 8 | 20 |
| Relationships with family or friends | 3 | 3 | 2 | 8 |
| Digital well-being | 0 | 1 | 0 | 1 |

**Table S2.** Survey questions about the alignment of Wellby with students’ well-being goals.

| Question | Response option | n (%) |
| --- | --- | --- |
| How well do you feel the wearable aligns with what you and your classmates asked for in the co-design session? | Extremely well | 10 (42%) |
|  | Somewhat well | 11 (46%) |
|  | Neutral | 3 (12%) |
|  | Somewhat not well | 0 (0%) |
|  | Not well at all | 0 (0%) |
| How well do you feel the app aligns with what you and your classmates asked for in the co-design session? | Extremely well | 13 (54%) |
|  | Somewhat well | 7 (29%) |
|  | Neutral | 4 (17%) |
|  | Somewhat not well | 0 (0%) |
|  | Not well at all | 0 (0%) |
| How well do you feel the Wellby system (the app and wearable) aligns with student's well-being needs in general? | Extremely well | 16 (55%) |
|  | Somewhat well | 6 (21%) |
|  | Neutral | 6 (21%) |
|  | Somewhat not well | 1 (3%) |
|  | Not well at all | 0 (0%) |

**Table S3.** The total engagement counts of student participants with Wellby app features.

| Feature | Total clicks |
| --- | --- |
| Customize app color | 417 |
| View resource topic | 128 |
| View HRV summary | 80 |
| Open breath pacer | 61 |
| Open to do list | 61 |
| View check-in summary | 58 |
| Open image resource | 55 |
| Save resource | 37 |
| Open video resource | 19 |
| Open website resource | 11 |
| Toggle off daily quote | 4 |
